# The role of prediction error and memory destabilization in extinction of cued-fear within the reconsolidation window

**DOI:** 10.1038/s41386-018-0299-y

**Published:** 2018-12-20

**Authors:** Emma N. Cahill, Melissa A. Wood, Barry J. Everitt, Amy L. Milton

**Affiliations:** 1 Department of Physiology, Development and Neuroscience, University of Cambridge, Cambridge, CB2 3EG UK; 2 Behavioural and Clinical Neuroscience Institute, Cambridge, CB2 3EB UK; 3 Department of Psychology, University of Cambridge, Cambridge, CB2 3EB UK

**Keywords:** Fear conditioning, Psychology

## Abstract

Extinction of a cued-fear memory within the reconsolidation window has been proposed to prevent fear reacquisition by reconsolidation interference. This ‘retrieval-extinction’ procedure has received interest for its therapeutic potential to reduce the impact of fear memories on behavior. To fully exploit its therapeutic potential, it is critical to understand the mechanisms that underlie the ‘retrieval-extinction’ effect. If the effect depends upon reconsolidation of the original memory, then it would be predicted that destabilization, induced by prediction error, would be critical for observing the effect. Here, the dependency of the retrieval-extinction effect on memory destabilization or prediction error was investigated in pavlovian cued-fear conditioned adult male rats. The requirement for memory destabilization, and thus reconsolidation, for the retrieval-extinction effect was subsequently investigated using region-specific pharmacological blockade of dopamine D1-receptors. Intra-basolateral amygdala antagonism of dopamine D1-receptors did not prevent the reacquisition of fear associated with the retrieval-extinction procedure. The requirement for prediction error was assessed by using a reinforced or non-reinforced memory retrieval trial before extinction, compared to a no-retrieval, extinction-only control. Both the reinforced (no prediction error) and non-reinforced retrieval sessions led to a decrease in fear reacquisition, suggesting that engagement of prediction error does not influence the occurrence of retrieval-extinction. Together, these data suggest that retrieval-extinction does not require memory destabilization, since behavioral or pharmacological interventions that prevent destabilization did not disrupt any capacity to attenuate fear.

## Introduction

Behavioral responses to conditioned stimuli (CSs) can be reduced through extinction training or the manipulation of memory reconsolidation. Both approaches are used to treat anxiety disorders such as post-traumatic stress disorder, but each has limitations. Extinction involves the learning of a new, inhibitory ‘CS-no fear’ memory through repeated CS exposure without the aversive outcome, allowing the new memory initially to outcompete the original ‘CS-fear’ memory for control over behavior (Pavlov, 1927, [1]). However, extinction training is associated with the risk of fear memory return through renewal, reinstatement, reacquisition, or spontaneous recovery of the original ‘CS-US’ association [2, 3]. By contrast, pharmacological blockade of memory restabilisation is hypothesized to persistently weaken or even erase the original memory [4]. Disruption of reconsolidation depends critically upon the induction of memory destabilization [5, 6], with the specific conditions likely depending on factors including memory age and strength [7].

While reconsolidation-based interventions are appealing from a therapeutic perspective, the requirement for pharmacological disruption of the memory poses challenges for translation. Behavioral interventions may be more translationally tractable: for example, exploiting the updating function of reconsolidation by conducting extinction training within the ‘reconsolidation window’ [8]. This ‘extinction within the reconsolidation window’ (or ‘retrieval-extinction’) procedure produced long-term reductions in fear that did not spontaneously recover, renew, or reinstate, with reduced subsequent reacquisition. Since 2009, studies have investigated retrieval-extinction as a means to persistently reduce fear or drug memories, albeit with varying degrees of success [9–13].

Despite some characterization of the underlying molecular mechanisms, it remains unclear whether retrieval-extinction depends upon reconsolidation. Monfils et al. [8] attributed the effect to extinction training updating the original memory with the new (safety) memory as it had become destabilized at reactivation, but the requirement for destabilization was not directly tested. This can be measured using at least two independent methods: one pharmacological and one behavioral. Thus, following non-reinforced CS presentation, dopamine neuron activation or dopamine release signals prediction error in both appetitive [14, 15] and aversive tasks [16], and increases in amygdala dopamine levels have been reported during the recall of conditioned fear memories [17]. Destabilization could be pharmacologically blocked by antagonism of the dopamine receptors required for signaling prediction error. Furthermore, antagonizing the GluN2B-subtype of NMDA receptor prevents fear memory destabilization [6, 18]. Thus, targeting these neurochemical systems should reveal whether retrieval-extinction depends upon memory destabilization. Behaviorally, memory destabilization is hypothesized to be induced by a violation of expectations at reactivation [19], neurally encoded as ‘prediction error’. Thus, a reactivation session in which there is no prediction error (because the CS is reinforced with shock) should not induce destabilization of the fear memory, and the ‘retrieval-extinction’ effect should not be observed [20].

Here, we used both pharmacological and behavioral approaches to test the hypothesis that memory destabilization is required for the retrieval-extinction effect. It was predicted that extinction within the reconsolidation window should prevent reacquisition of cued fear, and that blockade of destabilization would prevent the effect.

## Materials and methods

### Subjects

Subjects were 148 adult male Lister-Hooded rats (Charles River) weighing 250–300 g at the start of experiments. All animals were kept under a 12 h light/dark cycle (lights off at 7:00 A.M.) and were provided with food and water ad libitum, except for during behavioral procedures. Animals were housed in groups of four, unless surgery was performed after which they were pair housed. This research was conducted on Project Licence 70/7548 and has been regulated under the Animals (Scientific Procedures) Act 1986 Amendment Regulations 2012 following ethical review by the University of Cambridge Animal Welfare and Ethical Review Body (AWERB).

### Surgeries

Rats were anaesthetized with ketamine hydrochloride (100 mg/kg; Ketaset, Fort Dodge Animal Health) and xylazine (9 mg/kg; Rompun, Bayer), and implanted with a 22 gauge stainless steel bilateral indwelling guide cannula (Plastics One) aimed at the BLA. The coordinates were AP −2.6 mm and +4.5 mm lateral to the mid-line (relative to bregma), and −5.6 mm ventral to dura mater. Animals were injected subcutaneously with the analgesic carprofen (Rimadyl 5 mg/kg, Henry Schein Animal Health) at the end of surgery. Stainless steel obturators were inserted to maintain patency during recovery and in between infusions. All animals were given a minimum of 5 days to recover from surgery before beginning behavioral experiments.

### Intracranial drug infusions

Infusions were performed using a syringe pump (Harvard Apparatus, Edenbridge, UK) and 5 μl Hamilton syringes connected to injectors (28 gauge, projecting 4 mm beyond the guide cannulae) by polyethylene tubing. Before behavioral testing, rats were habituated to the infusion procedure by the bilateral administration of 0.25 μl of sterile saline solution per hemisphere (0.25 μl/min). Drugs or vehicles were administered (0.25 μl/min) in a volume of 0.5 μl per hemisphere immediately before the reactivation session. All infusions were begun 30 s after the injectors were inserted, and injectors were left in place for 30 s after the infusion ended to allow diffusion away from the injection site. For Experiment 1, the D1R receptor-selective antagonist SCH-23390 (SCH, Tocris Bioscience, Bristol, UK) was dissolved in sterile saline solution at a concentration of 4 μg/μl, as this dose has previously been shown to disrupt memory expression and destabilization [14, 21].

### Behavioral procedures

All procedures were conducted during the rats’ dark cycle. Rats were individually habituated to the conditioning boxes (Paul Fray Limited) for 2 h. On the training day, rats were placed in the box and after 25 min received an auditory CS presentation (60 s clicker, 10 Hz, 80 dB) that was coterminous with the presentation of the unconditioned stimulus (US), a scrambled footshock (0.5 mA, 0.5 s) delivered through the grid floor. Training consisted of three CS–US presentations with an inter-trial interval (ITI) of 5 min, except for Experiment 3 where rats received four CS-US presentations followed by another either three or four CS-US pairings the following day depending on group allocation (Fig. 3). Twenty-four hours later, the rats were either returned to the box for memory reactivation (Ret) or they remained in the homecage (No Ret) as controls. All experiments were run in two replicates. In Experiments 2 and 3, rats received an unreinforced CS presentation (Ret−) or a reinforced CS presentation (Ret+) 4 min after entering the chamber and remained there for 2 min after the CS presentation. In Experiment 4, the Cxt-Ext group were placed in the context for an equivalent period of time for the Ret session and returned to their homecage before extinction. The Ret-Cxt group had the same context exposure during the extinction session as the other groups, without any CS presentations. In Experiments 1 and 4, the retrieval groups had the same non-reinforced CS presentation (Ret). For all experiments, 1 h after reactivation all rats underwent extinction training. Animals received 17-20CS presentations depending on experiment and group (see Results for details). In experiment 2, due to recording equipment failure the extinction data for 12 animals was lost. Remaining animals are shown; (NoRet *n* = 6, Ret− *n* = 8, Ret+ *n* = 10). For all experiments, 24 h after extinction training, there was a reacquisition of fear session (ReAcq) in which rats were presented after 4 min in the box with the CS, coterminous again with shock. This session also served as a test of extinction memory. The following day, the extent of Reacquisition was tested and long-term memory (LTM) by a non-reinforced presentation of the CS. At the end of behavioral testing, rats were killed by exposure to a rising concentration of carbon dioxide, brains were collected in neutral buffered formalin (10%) and cannula locations checked using cresyl violet staining (for details see ref. [14]). Rats with placements outside of the BLA were excluded from all analyses.

### Behavioral assessment

All training, extinction and test sessions were video recorded for off-line behavioral analysis. The percentage of time freezing (absence of movement except for breathing) during the 1 min before (Pre CS) and during the 1-min CS was manually scored every 5 s from the videos by observers blind to the treatment. Statistical analyses, repeated-measures ANOVA, and planned LSD comparisons for three groups or Sidak comparisons for more than three groups were made using IBM SPSS Statistics 25. Where Mauchly’s Test of Sphericity indicated the assumption of sphericity had been violated, degrees of freedom were Greenhouse-Geisser corrected. Graphs and schemas were generated in GraphPad Prism 7.01 and Adobe Illustrator CS6. A total of 16 of the 148 animals were excluded from analyses because of inaccurate cannula placements or equipment failure.

## Results

### Antagonism of intra-BLA D1R signaling does not prevent the retrieval-extinction effect

**Fig. 1:**
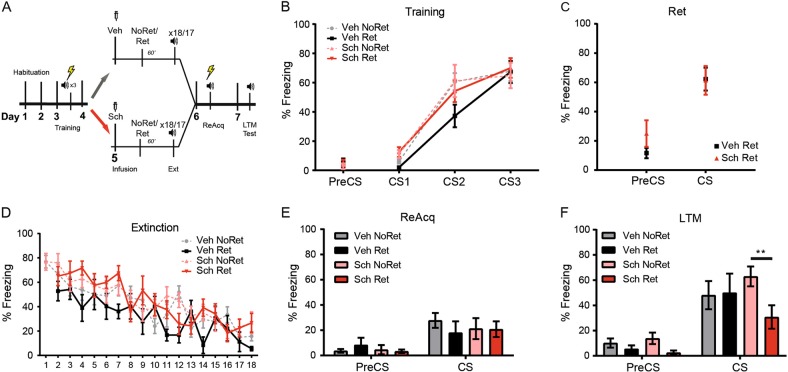
**a** Rats were divided into four groups, two receiving vehicle (Veh) infusion prior to either memory retrieval (Ret) or returned to the home cage (NoRet), and two receiving the D1R antagonist Schering-23390 (Sch) before retrieval or returned to home cage. **b** All groups acquired fear conditioning across CS-US presentations to an equal extent. **c** There was no difference in freezing between the two Ret groups, suggesting acute Sch infusion into the BLA had no effect on freezing expression itself. **d** All groups extinguished responding to the CS over time. **e** Following extinction all groups had reduced fearful behavior to the CS to an equivalent level. At the end of the CS the US was presented again for fear reacquisition (ReAcq). **f** The effects of reactivation (Ret) and/or Sch on the long term memory (LTM) of fear were tested 24 h later. Only the groups pre-treated with Sch appeared to have a retrieval-extinction effect on fear reacquisition. **p < 0.001

Based on the potential recruitment of dopaminergic signaling for aversive learning [16] and the proposed role of the BLA in unsigned neural encoding of prediction error [22], we targeted the BLA dopamine D1Rs to test whether they were required for the retrieval-extinction effect (Fig. 1a). All rats acquired conditioned fear (CS: *F*(_1.99, 53.8_) = 162.3, *P* < 0.0001, *ɲ*^2^ = 0.86) with no difference between the prospective experimental groups (Group: *F* < 1). The rats were allocated into four groups: the rats were administered either Vehicle (Veh) or the D_1_-selective dopamine receptor antagonist SCH-23390 (Sch) directly into the BLA before a retrieval trial (‘Veh Ret’ and ‘Sch Ret’ groups) or before return to home cage (‘Veh NoRet’ and ‘Sch NoRet’). This design allowed the assessment of the acute effect of Sch administration on the expression of cued-fear (Fig. 1c), which revealed no differences between groups in fear expression (Drug: *F* < 1). One hour later all rats underwent extinction (Fig. 1d). Animals received 17 or 18 CS presentations. One cohort from both the Ret groups (*n* = 4/group) received an ‘extra’ CS during extinction (18CS) due to experimenter error, but these animals were not excluded from the experiment as their freezing did not significantly differ from any other groups neither during extinction nor on the following day (ReAcq); moreover their inclusion or exclusion did not alter the final conclusion of this experiment. For comparison across groups, the freezing across time for 17 CSs (CS2 to CS18; CS1 was the previous retrieval trial) was considered. All animals extinguished responding to the CS over time (CS: *F*(_8.61, 189.5_) = 11.5, *P* < 0.0001, *ɲ*^2^ = 0.34). Overall, drug infusion had a significant effect on freezing across extinction (Drug: *F*(_1,22_) = 7.136, *P* = 0.014, *ɲ*^2^ = 0.25), yet there were no differences between the groups that had undergone retrieval and those that had not (React: *F*(_1, 22_) = .091), and there was no significant interaction (Drug*React: *F*(_1,22_) = 3.107, *P* = 0.092). Moreover, when the last three CS presentations were analyzed across groups there was no effect of drug treatment (Drug: *F*(_1,30_) = 2.67, *P* = 0.113) nor reactivation (React: *F* < 1). The following day, all groups had low levels of freezing to the CS regardless of drug treatment (Drug: *F* < 1) or retrieval (React: *F*(_1,31_) = 1.06, *P* = 0.453, *ɲ*^2^ = 0.01). At a memory retention test 24 h later (Fig. 1f), all animals showed greater freezing to the CS than the PreCS period (*F*(_1,30_) = 50.1, *P* < 0.0001, *ɲ*^2^ = 0.63), but the omnibus ANOVA revealed no main effect of drug treatment (Drug: *F* < 1), an effect of retrieval (React: *F*(_1, 30_) = 4.24, *P* = 0.048, *ɲ*^2^ = 0.12), and no Drug×CS interaction (Drug×React: *F*(_1, 30_) = 3.28, *P* = 0.08, *ɲ*^2^ = 0.10). As our a priori prediction was that an impairment in fear reacquisition would be observed in the Veh Ret group as compared to the Veh NoRet group, but not the Sch-treated groups, we also compared the freezing of the Veh- and Sch-treated groups separately. However, contrary to our hypothesis, Sidak-corrected pairwise comparisons showed that Sch Ret animals froze less than Sch NoRet (*P* = 0.030) whereas there was no difference in fear reacquisition between the Veh Ret and Veh NoRet groups (*P* = 0.909). We also used an alternative approach, targeting the GluN2B-subtype of NMDA receptors that are required for destabilization to occur using the antagonist ifenprodil (see supplementory data, [6, 18]). In this experiment, again there was no retrieval-extinction effect observed in the vehicle groups. The Ifen Ret group had numerically lower freezing reacquisition than the Ifen NoRet group, which would support the findings of Experiment 1, however this did not reach significance (see supplementary figure 1). Overall, this counterintuitive result suggests that rather than activity at BLA D1Rs being necessary for retrieval-extinction, in fact antagonism at D1Rs facilitates the impairment in fear memory reacquisition associated with retrieval-extinction, which is otherwise not observed in the vehicle-treated animals.

### Prediction error does not influence the effect of retrieval-extinction to lessen fear reacquisition

**Fig. 2:**
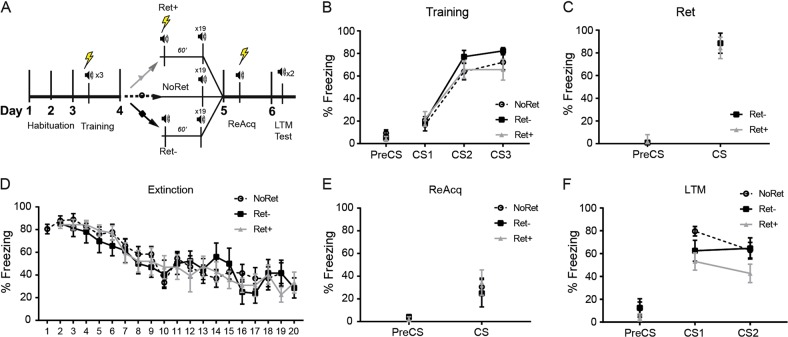
**a** Schema of experimental protocol. Animals were divided into three groups after training: no retrieval controls (NoRet), reinforced retrieval (Ret+), or non-reinforced retrieval (Ret−). **b** All groups acquired fear conditioning to a similar level across three CS-US pairings during training. **c** The Ret− and Ret+ groups had similar freezing levels during memory retrieval (Ret), for the Ret+ group this CS presentation terminated with the US. **d** All animals were presented with 19 CSs to extinguish the fear memory. **e** Following extinction all groups had reduced freezing to the CS to an equivalent level across groups. During the reacquisition (ReAcq) session the CS was presented again but coterminous with a foot shock to all groups. **f** The NoRet animals reacquired to the greatest extent, significantly more than the Ret + group

In Experiment 1, we did not observe the expected retrieval-extinction effect in our Veh-treated groups. For Experiment 2, the extinction training was extended to 19CSs and two presentations were given at test to mitigate against ceiling effects of freezing. Experiment 2 was designed to test whether explicitly not providing a prediction error (Fig. 2a), by reinforcing the reactivation session, would prevent the effect of retrieval-extinction on fear reacquisition (i.e. whether extinction following a reinforced reactivation session would produce behavior equivalent to standard extinction training). All rats froze to the CS more following shock pairings during training (Fig. 2b, CS: *F*(_2.3,51.4_) = 91.97, *P* < 0.0001, *ɲ*^2^ = 0.81) with no differences between the prospective groups (Group: *F*(_2,22_) = 2.76). After training, the animals were split into three groups. The NoRet control group remained in the homecage whilst the Ret− and Ret+groups underwent a retrieval session during which they expressed equivalent levels of freezing to the CS (Fig. 2c, CS (_1,14_) = 1113.63, *P* < 0.0001, *ɲ*^2^ = 0.99, Group: *F* < 1). The Ret + group received an additional shock after CS presentation in this session. One hour later all groups underwent extinction training (Fig. 2d). All rats received 19 CS presentations. All groups extinguished during this session (CS: *F*(_9.3, 194.8_) = 18.9, *P* < 0.0001, *ɲ*^2^ = 0.47) with no differences between experimental groups (Group: *F*(_2,21_) < 1). The following day, during the reacquisition session, there was still a significant effect of the CS on freezing but freezing levels to the CS did not differ between groups (Fig. 2e, CS: *F*(_1,22_) = 17.96, *ɲ*^2^ = 0.45, Group: *F* < 1). All groups received a shock after the CS presentation in this session. The next day freezing to the CS was tested with two CS presentations. All groups showed freezing to the CS (CS: *F*(_2,44_) = 63.093, *P* = 0.001, *ɲ*^2^ = 0.741). Freezing during the CS differed between the experimental groups (Group: *F*(_2,22_) = 4.104, *P* = 0.031, *ɲ*^2^ = 0.272). LSD pairwise comparisons revealed that freezing was higher in the NoRet group than the Ret + group (*P* = 0.014), but not significantly for the Ret− group (*P* = 0.098). However, there were no statistical differences in freezing between the Ret+  and Ret− groups (*P* = 0.368). Contrary to expectations, the Ret+ group did not reacquire fear to the same extent as NoRet controls, i.e. there was a reduction in the reacquisition of fear in the Ret + group, equivalent to the retardation of fear reacquisition seen in the classical ‘retrieval-extinction’ Ret− control group. This indicates that prediction error is not necessary to observe the effect of retrieval-extinction on the subsequent reacquisition of fear.

**Fig. 3:**
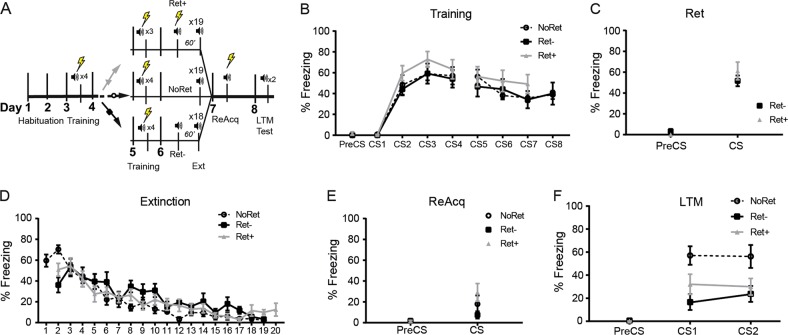
**a** Schema of experimental protocol. Rats were divided into three groups. NoRet and Ret− receiving 8 US-CS pairings (4 per session across 2 days), whereas the Ret + group received 7 US-CS pairings across training (4 in first session and 3 in the second). **b** All groups acquired fear conditioning to a similar level across pairings during training. The NoRet and Ret group received 8 pairings, whilst Ret+ received 7, to equate for CS-US pairing exposure across days. **c** The Ret− and Ret+ groups had similar freezing levels during memory reactivation (Ret). **d** All groups extinguished fear over the session. NoRet and Ret+ groups were presented with 19 CSs whilst Ret− were presented with 18 CSs to equate for CS exposure across days. **e** Following extinction all groups had reduced fearful behavior to the CS to an equivalent level. During the reacquisition (ReAcq) session the CS was presented again coterminous with a footshock to all groups. **f** The NoRet animals reacquired freezing to the greatest extent, significantly more than the Ret− group and the Ret+ group

Similar to our finding with pharmacological blockade of destabilization (Experiment 1), behavioral occlusion of prediction error seemed to enhance the retrieval-extinction effect. However, one potential alternative explanation for the impairment in reacquisition in the Ret+ group is that prediction error was, in fact, engaged during the reinforced re-exposure session because the initial fear training did not result in asymptotic learning. In order to discriminate between this hypothesis and a lack of requirement for prediction error in the ‘retrieval-extinction’ effect, Experiment 3 was designed to ensure asymptotic fear learning by doubling the number of CS-US pairings during training. Therefore, both the NoRet and Ret− groups received 8 CS-shock pairings over two days, while the Ret + group received 7 CS-shock pairings during training, to equate for the additional shock they would receive in the re-exposure session the following day (Fig. 3a). All groups acquired fear conditioning to a similar level across pairings during training (Fig. 3b) when conditioned freezing was compared up to the 7^th^ CS-US pairing (CS: *F*(_3.84,65.3_) = 50.5, *P* < 0.0001, *ɲ*^2^ = 0.75) with no difference between groups (Group: *F* < 1). The following day (Fig. 3c), the Ret+ and Ret− groups showed similar levels of freezing to the CS (CS: *F* < 1) indicating that learning was asymptotic by the seventh CS. All groups extinguished over time (CS: *F*(_6.6, 131.1_) = 40.6, *P* < 0.0001, *ɲ*^2^ = 0.67) with no difference between the groups (Group: *F* < 1). During reacquisition (Fig. 3e), freezing was equivalent across groups (Group: *F*(_2,21_) = 2.05, *P* = 0.153). However, as in Experiment 2, when reacquisition was assessed on the test day (LTM, Fig. 3f), the NoRet animals had reacquired fear to the greatest extent, with no differences between the Ret+ and Ret− groups. There was a significant difference between groups (Group: *F*(_1,21_) = 71.191, *P* = 0.001, *ɲ*^2^ = 0.772). A LSD analysis revealed that freezing was significantly different from the NoRet group for Ret− group (*P* = 0.002), and for the Ret+ group (*P* = 0.038). However, there was no significant difference in freezing between the Ret− and Ret+ group (*P* = 0.185). Taken together, these two experiments indicate that the reduction in the reacquisition of fear produced by the retrieval-extinction procedure does not rely on the induction of prediction error during the retrieval trial.

### Context exposure alone does not evoke the retrieval-extinction effect

**Fig. 4:**
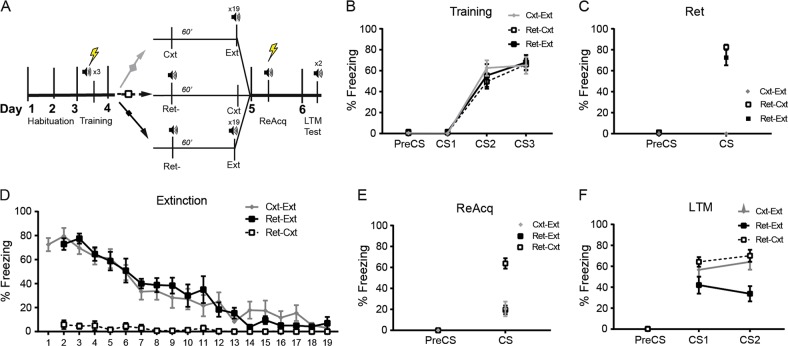
**a** Rats were divided into three groups after training. **b** The prospective groups acquired fear conditioning to the same extent. **c** The Ret groups were re-exposed to an unreinforced presentation of the CS, and the Cxt group was placed in the experimental context for the same duration of time. **d** The Ext groups (Cxt-Ext and Ret-Ext) extinguished across the session whereas the Ret-Cxt group were placed in the experimental context for the same duration of time. **e** The groups that underwent extinction presented low levels of freezing the following day, in contrast to the Ret-Cxt group. At the end of the session all groups received a foot shock coterminous with the CS presentation. **f** The Ret-Ext group reacquired freezing to a lower extent than the Cxt-Ext or Ret-Cxt groups, indicating that CS presentation, and not just context exposure, is needed for the effect

A potential explanation for the pattern of results described above is that both Ret+ and Ret− groups were re-exposed to the fear context for equivalent amounts of time, and for longer than the NoRet groups. In order to determine whether CS reactivation per se is required for the retrieval–extinction effect, Experiment 4 was designed to control for context exposure during the retrieval and extinction sessions. All groups acquired a freezing response to the CS across training (CS: *F*(_1.889,63_) = 129.134, *p* < 0.0001, *ɲ*^2^ = 0.86), with no difference between the prospective groups (Group: *F*(_2,21_) = 0.262, *p* = 0.262, *ɲ*^2^ = 0.024). For the retrieval session the Cxt-Ext group was placed in the context without CS presentations, and as expected there was a significant difference in freezing across groups at the CS time point (Group: *F*(_2,21_) = 100.572, *p* < 0.0001, *ɲ*^2^ = 0.905). The next day the Ret-Cxt group was placed in the context for the same duration as the extinction session (without CS presentations). CS exposure significantly decreased freezing across the extinction session for the Ret-Ext and Cxt-Ext groups (CS: *F*(_17,357_) = 39.963, *p* < .0001, *ɲ*^2^ = 0.656). The next day there was still an overall effect of CS presentation on freezing (CS: *F*(_1,21_) = 118.220, *p* < 0.0001, *ɲ*^2^ = 0.849) which differed across the groups as the Ret-Cxt group retained high levels of freezing (Group: *F*(_2,21_) = 21.028, *p* < 0.0001, *ɲ*^2^ = 0.667). On the test day (Fig. 4f), there was a significant effect of CS on freezing, (CS *F*(_2,42_) = 14.089, *p* < 0.0001, *ɲ*^2^ = 0.870) and a difference across the groups (Group *F*(_2,21_) = 6.229, *p* = 0.008 *ɲ*^2^ = 0.372). At CS1, the Ret-Cxt group, which had experienced no extinction training, displayed the highest levels of freezing as expected, significantly more than the Ret-Ext group (Ret-Cxt vs Ret-Ext *p* = 0.031), but not those that received extinction (Ret-Cxt vs Cxt-Ext *p* = 0.441). Freezing did not significantly differ between Ret-Ext and Cxt-Ext (*p* = 0.143) at CS1. At CS2, the Ret-Ext group’s level of freezing remained significantly lower than the Cxt-Ext group (*p* = 0.001), and also lower than the Cxt-Ext group (*p* = 0.005). Freezing did not differ between the Ret-Cxt versus Cxt-Ext *p* = 0.552. These results suggest that Ret-Ext still confers an advantage over standard extinction in diminishing or preventing fear relapse and independently of exposure to the context alone (Ret-Ext versus Cxt-Ext).

## Discussion

Since its initial demonstration, many studies were inspired to adapt the original procedure proposed by Monfils and colleagues to attenuate fear persistence using retrieval–extinction [8]. In spite of this growing literature, the mechanisms underlying the effect on relapse of cued fear have not been fully defined. Our results provide evidence that retrieval–extinction can operate in absence of the typical criterion for memory reconsolidation, namely prediction error and destabilization. We used intracerebral pharmacological and behavioral interventions to disentangle whether the retrieval–extinction effect depends on, or is influenced by, destabilization of memory or prediction error respectively. When rats received infusions of a dopamine D1R antagonist into the amygdala prior to retrieval, they displayed an intact retrieval-extinction effect. In addition, rats consistently showed a retrieval–extinction effect despite the absence of prediction error following reinforced CS memory retrieval. The effect was not seen if rats were simply exposed to the conditioning context prior to extinction, suggesting that the effect is driven by a CS-mediated process. Based on these findings, we hypothesize that retrieval–extinction can act through mechanisms that enhance the consolidation of extinction learning, independently of memory reconsolidation.

Reconsolidation and extinction have been suggested to be mutually exclusive events [19, 23], and thus preventing the engagement of one (here reconsolidation) might enhance the ability of the other to become the dominant memory process. The reduction of fear reacquisition following the pharmacological manipulations experiments could be interpreted as the prevention of destabilization of the original fear memory, via antagonism of either D1R or GluN2B-NMDAR, seeming to favor the retrieval-extinction effect. Our observations in Experiment 2 and also in Experiment 3, that even a reinforced retrieval trial was surprisingly effective in producing the reduced reacquisition of fear associated with retrieval–extinction further supports this hypothesis. Learning reached asymptote, yet both reinforced and non-reinforced retrieval conditions impaired fear reacquisition at subsequent test. This suggests that the retrieval–extinction effect does not require a prediction error to occur at retrieval.

If retrieval is driving extinction in this case, then why are the animals resistant to reinstatement? One explanation may be that the retrieval session (reinforced or not) sufficiently engages and primes the consolidation of extinction driven by any further CS presentations within an appropriate timeframe. The temporal window for the consolidation of extinction is thought to be similar in duration to the reconsolidation window; therefore manipulations outside that 6-hour window would not affect the consolidation of extinction. Consistent with this view are reports that DCS-enhanced fear extinction became resistant to renewal and reinstatement [24, 25] although fear reacquisition did not [26].

There is extensive evidence from crabs to rats to humans that pavlovian memory destabilization occurs when there is a mismatch between what is expected and what actually occurs [19, 20]. Notably retrieval-extinction for contextual fear memories has been shown to require a prediction error [27, 28]. After determining the amount of contextual re-exposure needed to induce memory destabilization (as shown by a subsequent reduction in fear produced by midazolam administration), Pineyro et al. [27] demonstrated that only this same exposure time followed by extinction prevented fear reacquisition. It was further demonstrated that inhibiting destabilization using nimodipine prevented the impaired reacquisition of contextual fear produced by prior retrieval–extinction [28]. On the other hand, in that same study retrieval–extinction of cued-fear surprisingly resulted in levels of freezing that were significantly higher in the retrieval–extinction group versus normal extinction at memory test 24 h after the manipulation. Taken together, the case for effective retrieval–extinction being dependent on destabilization is stronger for contextual than for cued fear.

Why then might cued fear not require prediction error for the retrieval–extinction effect? It is difficult to directly compare findings from cued and contextual fear procedures, but in general contextual protocols produce lower freezing levels than discrete cues (for example, ref. [28]). Thus, differences in the strength of the original memory may regulate whether prediction error engages updating or new learning (i.e. extinction). It has been found that the boundary conditions that determine whether reconsolidation occurs vary depending on the strength and age of the memory [7]. This view is supported by evidence from contextual fear conditioning where the length of time between training and test (i.e. the age of the memory) influenced whether reconsolidation or extinction took place after re-exposure [29]. This study showed that older memories were stronger than the younger ones at test. This supports older/stronger memories treating a prediction error episode as new learning, rather than updating. In the case of retrieval–extinction of cued fear memories, this would suggest that the decreased fear observed because a strong memory resists updating and pushes instead the information into new extinction learning.

Importantly, multiple mechanisms may underlie the effect of retrieval–extinction and these may vary across experimental procedures. However, the data presented here support an interpretation of the retrieval–extinction effect that is more consistent with the enhancement of extinction mechanisms than with the engagement of memory destabilization and reconsolidation processes. Different levels of success with this procedure have been reported for aversive and appetitive memories [12, 30]. Based on the literature, and our present findings, it remains debatable whether retrieval–extinction is a reproducible effect across different tasks. In the first experiment, it is difficult to reconcile why there was no retrieval–extinction effect in the vehicle-treated group. The major difference between the first and subsequent experiments was the cannulation surgery to target the BLA, however there is no evidence of any behavioral change in the rate of learning or extinction in these animals when compared to uncannulated animals that could suggest a change in baseline performance. The housing of animals was reduced to two per cage, versus four in the subsequent experiments, however recent meta-analyses suggest a smaller cage group should in fact enhance the effect [10]. Moreover, in the Sch-treated animals the Ret group showed impaired reacquisition, which argues against a simple explanation of the difference being due to surgery effects. Another pharmacological approach, using ifenprodil to target GluN2B-NMDARs, produced similar findings (Fig S1). Most discouragingly a direct replication attempt was recently published that did not find any evidence for the reduced return of fear relative to standard extinction training [31]. Without a better understanding of how retrieval–extinction works, or why it does not work in some circumstances, any attempts to translate the protocol to clinical populations are likely to remain suboptimal. Our present results, combined with previous studies, emphasize the importance of defining the mechanisms underlying the retrieval–extinction effect in order to refine the protocols used to study it as well, as facilitating translation to the clinic in the treatment of psychiatric disorders characterized by maladaptive memories.

## Funding and disclosure

This work was funded by a UK Medical Research Council program grant (no. G1002231) awarded to BJE and ALM and BBSRC Anniversary Future Leaders Fellowship (BB/M01407X/1) awarded to ENC. MAW was supported by the Cambridge Australia Poynton Scholarship, Overseas Research Scholarship and Oon Khye Beng and Ch’hia Tsio Trust Fund. ALM is the Ferreras-Willetts Fellow in Neuroscience at Downing College, Cambridge. ENC is currently receiving funding from Boehringer Ingelheim Pharma GmbH & Co. for a project unrelated to this work.

## Supplementary information

Supplementary Fig 1

Supplementary Fig 2

Supplementary Information
