# Supplementary figures and images for: The role of prediction error and memory destabilization in extinction of cued-fear within the reconsolidation window
Source: Neuropsychopharmacology. 2018 Dec 20;44(10):1762–8. doi: 10.1038/s41386-018-0299-y (PMC6699995; doi:10.1038/s41386-018-0299-y)

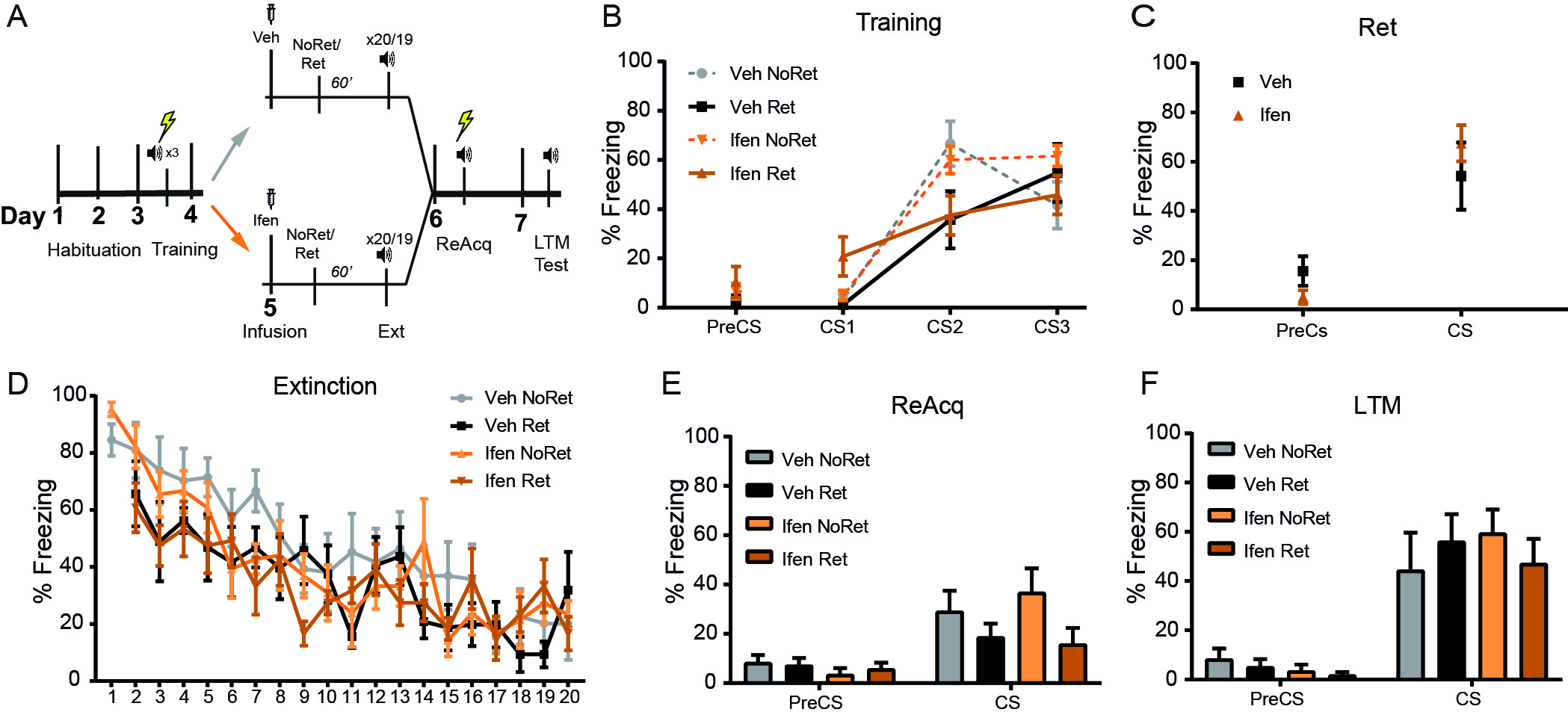

Supplement: Supplementary file 1 — Supplementary Fig 1 [file 41386_2018_299_MOESM1_ESM.jpg]

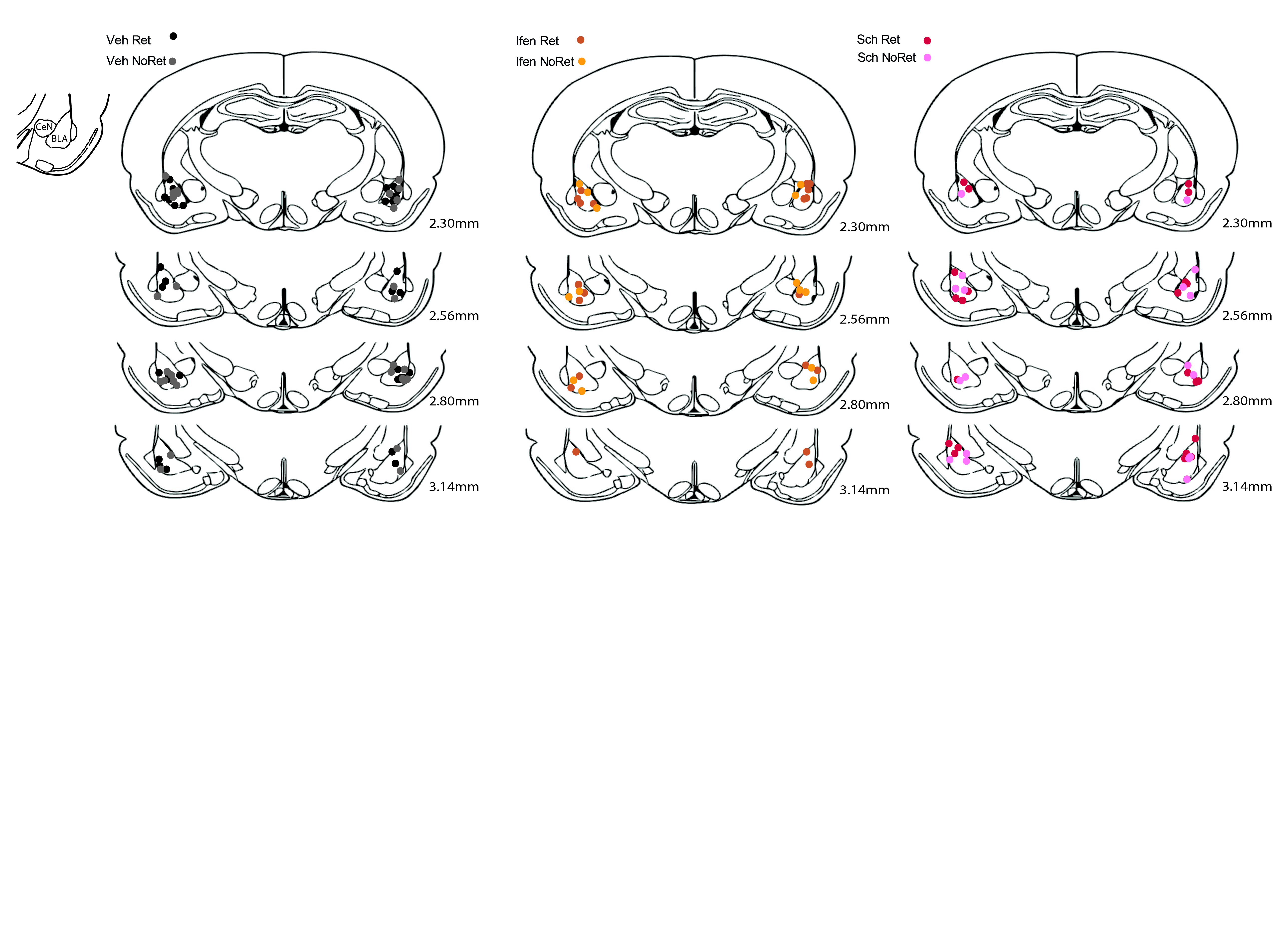

Supplement: Supplementary file 2 — Supplementary Fig 2 [file 41386_2018_299_MOESM2_ESM.jpg]
