# Supplementary material for: The role of prediction error and memory destabilization in extinction of cued-fear within the reconsolidation window
Source: Neuropsychopharmacology. 2018 Dec 20;44(10):1762–8. doi: 10.1038/s41386-018-0299-y (PMC6699995; doi:10.1038/s41386-018-0299-y)
Supplement: Supplementary file 3 — Supplementary Information [file 41386_2018_299_MOESM3_ESM.docx]

Supplementary Information

**Supplementary Methods:**

Infusions were as described in the main methods. For Experiment 4, the GluN2B diheteromeric receptor-selective (Williams, 1993) NMDAR antagonist ifenprodil (Ascent Scientific) was dissolved in PBS at a concentration of 2μg/μl, this dose has previously been shown to disrupt memory destabilization (Ben Mamou et al., 2006, Milton et al., 2013).

**Antagonism at BLA GluN2B containing NMDARs does not block the retrieval extinction effect**

The results of Experiment 1 were counterintuitive, as we failed to replicate the impaired fear memory reacquisition reported by others and that had been observed in uncannulated animals in our Experiments 3 and 4, and treatment with a dopamine receptor antagonist, which should interfere with signaling of prediction error, appeared to facilitate the impairment in fear memory reacquisition produced by the retrieval-extinction procedure. As these results were unexpected, we used an alternative approach to assess whether memory destabilization is necessary for the retrieval-extinction effect, targeting the GluN2B-subtype of NMDA receptors that is required for destabilization to occur (Ben Mamou et al., 2006; Milton et al., 2013). Experimental procedures and methods were similar to Experiment 1, except that animals received either Vehicle (‘Veh Ret’ and ‘Veh NoRet’ groups) or the GluN2B-selective NMDA receptor antagonist ifenprodil (‘Ifen Ret’ and ‘Ifen NoRet’). All groups acquired fear conditioning (Fig 4B) across CS-US presentations to an equal extent (CS: F(_1.8, 30.95_) = 59.6, P<0.0001, ɲ^2^=0.78) with no differences between the prospective experimental groups from drug treatment (Drug: F<1) or reactivation (React: F(_1, 17_) = 1.01, P=0.329) nor any interaction (Drug*React: F<1). As before, Ifenprodil or appropriate Vehicle was administered by microinfusion directly into the BLA and animals immediately placed in the experimental chamber (Ret groups) or the home cage (NoRet groups). Both Veh Ret and Ifen Ret groups froze equally to the CS presentation (CS: F(_1, 16_) =42.43, P<0.0001, ɲ^2^=0.73) with no effect of the Ifenprodil infusion versus Vehicle on freezing expression (Drug: F<1). One hour later, all animals underwent extinction training (Fig 4D). For Experiment 4, animals received 19 or 20 CS presentations. For comparison across groups during extinction, the freezing across time for 17 CSs (CS2 to CS18) was considered. All groups extinguished fear across the session (CS: F(_17, 476_) = 13.8, P<0.0001, ɲ^2^=0.33), with no effect of drug treatment (Drug: F<1) but slightly better extinction in the two Ret groups (React: F(_1, 28_) = 4.44, P=0.044, ɲ^2^=0.14) with no interaction (Drug*React: F(1,28) = 0.951). However, when the last three CS presentations were compared, there was neither an effect of drug treatment (Drug: F(_1,28_) = 1.98, P=0.170) nor reactivation (React: F <1). The day after extinction (Fig 4E), all groups froze more to the CS than during the PreCS period (CS:F(_1,27_) = 30.2, P<000.1, ɲ^2^=0.53), but with no differences between the Ret and No Ret groups (React: F(_1,27_) = 2.62, P=0.117) and no differences between Veh and Ifen groups (Drug: F < 1). The effects of reactivation and/or Ifen on the reacquisition of fear after the shock-CS exposure were tested 24h later (LTM, Fig 4F). All animals froze more to the CS than during the PreCS period (CS: F(_1,27_) = 75.4, P<0.0001, ɲ^2^=0.74), but with no main effect of drug treatment (Drug: F<1) or reactivation (React: F< 1) and no interaction (Drug*React F < 1). Sidak-corrected pairwise comparisons showed no significant difference between Veh NoRet and Veh Ret (P = 0.490) nor Ifen NoRet and Ret (P = 0.614). In this experiment, again there was no retrieval-extinction effect observed in the vehicle groups. The Ifen Ret group had lower freezing reacquisition than the Ifen NoRet group, which would support the findings of Experiment 1, however this did not reach significance.

**Legends:**

**Supplementary Figure 1**

A: Rats were divided into four groups as in Experiment 1. Two groups received vehicle infusion prior to retrieval (Ret) or were returned to homecage (NoRet) and the other two groups received the GluN2B-preferring antagonist Ifenprodil (Ifen).

B: All groups acquired fear conditioning across CS-US presentations to an equal extent.

C: Both groups significantly froze in response to the CS presentation at retrieval, with no effect of Ifen on freezing expression.

D: All groups extinguished fear across the session.

E: All groups had low levels of freezing to the CS during the reacquisition session.

F: All groups reacquired fear the following day at memory test.

**Supplementary Figure 2**

Representative image of the cannula placement for the infusion of vehicle (left), GluN2B-preferring antagonist ifenprodil (middle) and D1R antagonist Schering 23390 (right). Darker shades represent the placements for rats that were given a retrieval session prior to extinction and after the infusion, lighter shades (NoRet) were returned to the homecage post-infusion.
